# Evaluation of different formulas for LDL-C calculation

**DOI:** 10.1186/1476-511X-9-27

**Published:** 2010-03-10

**Authors:** Ana Vujovic, Jelena Kotur-Stevuljevic, Slavica Spasic, Nada Bujisic, Jelena Martinovic, Milica Vujovic, Vesna Spasojevic-Kalimanovska, Aleksandra Zeljkovic, Dragoljub Pajic

**Affiliations:** 1 Institute of Medical Biochemistry, Faculty of Pharmacy, Belgrade, Serbia; 2 "Belladonna" Clinical Chemistry Laboratory, Zemun, Belgrade, Serbia; 3 Belgrade Clinical Centre, Laboratory Department, Belgrade, Serbia; 4 "Sava Stanojevic" Health Centre, Trstenik, Serbia

## Abstract

**Background:**

Friedewald's formula for the estimation of LDL-C concentration is the most often used formula in clinical practice. A recent formula by Anandaraja and colleagues for LDL-C estimation still needs to be evaluated before it is extensively applied in diagnosis. In the present study we validated existing formulas and derived a more accurate formula to determine LDL-C in a Serbian population.

**Methods:**

Our study included 2053 patients with TG ≤ 4.52 mmol/L. In an initial group of 1010 patients, Friedewald's and Anandaraja's formulas were compared to a direct homogenous method for LDL-C determination. The obtained results allowed us to modify Friedewald's formula and apply it in a second group of patients.

**Results:**

The mean LDL-C concentrations were 3.9 ± 1.09 mmol/L, 3.63 ± 1.06 mmol/L and 3.72 ± 1.04 mmol/L measured by a direct homogenous assay (D-LDL-C), calculated by Friedewald's formula (F-LDL-C) and calculated by Anandaraja's formula (A-LDL-C), respectively in the 1010 patients. The Student's paired t-test showed that D-LDL-C values were significantly higher than F-LDL-C and A-LDL-C values (p < 0.001). The Passing-Bablok regression analysis indicated good correlation between calculated and measured LDL-Cs (r > 0.89). Using lipoprotein values from the initial group we modified Friedewald's formula by replacing the term 2.2 with 3. The new modified formula for LDL-C estimation (S-LDL-C) showed no statistically significant difference compared to D-LDL-C. The absolute bias between these two methods was -0.06 ± 0.37 mmol/L with a high correlation coefficient (r = 0.96).

**Conclusions:**

Our modified formula for LDL-C estimation appears to be more accurate than both Friedewald's and Anandaraja's formulas when applied to a Serbian population.

## Background

The concentration of low-density lipoprotein cholesterol (LDL-C) is one of the strongest markers of atherosclerosis and predictor for assessing coronary heart disease (CHD) risk. Strong positive association between increased LDL-C and CHD has been well documented [1-3]. The National Cholesterol Education Programme's (NCEP) Adult Treatment Panel III (ATP III) deemed that LDL-C concentration was the primary basis for treatment and appropriate patients' classification in risk categories [4] demonstrating that both accuracy and precision of LDL-C analysis are critically important.

The reference method for LDL-C concentration measurement, which combines ultracentrifugation-polianion precipitation, it is not readily available and also impractical in the routine laboratory [5]. A new generation of direct homogenous assays [6,7] for LDL-C determination in serum has been developed with a satisfactory degree of accuracy but at the same time they are expensive.

Despite some limitations, Friedewald's formula [8] is still the most commonly employed procedure in clinical laboratories for the estimation of LDL-C concentration and it has been considered acceptable for patients' classification [4]. It is often used in developing countries, including Serbia, due to its simplicity, convenience and low cost. The increase in cardiovascular disease in Serbian adults [9] together with the fact that risk factors need to be established at an early stage of disease underline the necessity to obtain the most precise and reliable formula for LDL-C calculation.

As already reported by other authors the homogeneous methods and Friedewald's formula are not capable of providing identical results [10,11]. Attempts have been made to evaluate and refine Friedewald's original formula. Recently, a new formula for LDL-C estimation was proposed by Anandaraja and colleagues [12] as a substitute for Friedewald's formula in the Indian population.

The present study was aimed to assess the validity of LDL-C values calculated by the Friedewald's formula (F-LDL-C) and those derived from Anandaraja and colleagues (A-LDL-C) and to compare them to values obtained by the direct method (D-LDL-C) in order to determine if a new formula could be applicable to a Serbian population. We also examined correlations and concentration differences obtained by the calculations and the direct method. The results of the present study were used to derive a new formula for calculation of LDL-C concentration (S-LDL-C) that appears to be more accurate than the latter two in a Serbian population. We also examined the classification concordance in relation to the NCEP ATP III LDL-C cut-off points between measured LDL-C and LDL-Cs derived by the three formulas to determine whether different calculation methods could affect patients' classification of heart disease risk.

## Materials and methods

### Participants

The first studied population (initial group) consisted of 1010 patients (51.9% male, mean age 53.7 ± 14.6 years) who underwent routine lipid status estimation as a part of a regular annual medical check-up. It was performed in the "Belladonna" Clinical Chemistry Laboratory during the whole of 2007. The second studied population (validation group) consisted of 1043 patients (42.9% male, mean age 54.2 ± 13 years) who were attending regular health check-ups in the "Sava Stanojevic" Health Centre between February and August 2008.

Blood samples were obtained in the morning after an overnight fast from all subjects and were analysed on the day of blood collection. Patients (45 in the initial group and 53 in the validation group) with triglyceride (TG) levels ≥4.52 mmol/L were excluded from further analysis. All the study participants were free of any confirmed renal, hepatic or cardiovascular disease and diabetes mellitus.

All participants gave informed consent prior to enrolment in our study that was planned according to the ethical guidelines laid down by the Declaration of Helsinki.

### Lipid-lipoprotein analyses

Total cholesterol (TC) and TG levels were measured enzymatically by CHOD-PAP and GPO-PAP methods (Roche Diagnostics GmbH, Mannheim, Germany), respectively according to the manufacturer's specifications. High-density lipoprotein cholesterol (HDL-C) was measured using a homogeneous assay without precipitation (Roche Diagnostics GmbH, Mannheim, Germany) [13].

A homogenous enzymatic colorimetric assay offered by Kyowa Medex and distributed by Roche Diagnostics, was used to measure LDL directly. The principle of D-LDL-C determination is as follows: At pH 6.75 and in the presence of magnesium ions, sulphated α-cyclodextrin and dextran sulphate the enzymatic reaction for cholesterol in very low-density lipoprotein (VLDL) and chylomicrons is markedly reduced. Polyoxyethylenepolyoxypropylene block polyether (POE-POP) blocks cholesterol, especially in HDL enabling LDL-C measurement by a conventional enzymatic reaction with cholesterol oxidase, cholesterol esterase and peroxidase (Roche Diagnostics GmbH, Mannheim, Germany) [6,13]. Homogeneous assay has been shown to meet current NCEP criteria for precision (CV < 4%), accuracy (bias < 4%) and for total analytical error (<12%) [4,13]. The intra-assay CVs for direct LDL-C were 1.8% at 2.0 mmol/L and 1.5% at 4.95 mmol/L and the inter-assay CVs were 2.3% at 1.27 mmol/L and 2.1% at 2.78 mmol/L.

All analyses in both laboratories were preformed on Roche Hitachi 911 Chemistry Analysers (Roche Diagnostics GmbH, Mannheim, Germany).

LDL-C concentrations were also calculated by Friedewald's formula [8]: F-LDL-C (mmol/L) = TC - HDL-C - TG/2.2 and by Anandaraja's formula [12] A-LDL-C (mg/dL) = 0.9*TC - 0.9*TG/5 - 28. Values in mg/dL were calculated and then expressed in mmol/L.

The percentage difference (%ΔLDL) defined as calculated LDL-C minus D-LDL-C compared to the direct measurement was calculated using the following formula: %Δcalculated LDL-C = [(calculated LDL-C)-(D-LDL-C)]/D-LDL-C*100. Our study evaluated the ability of the three formulas to correctly classify subjects into the risk categories given by NCEP ATP III using the D-LDL-C concentrations as the true values. These cut-off points were <2.59, 2.60 - 3.35, 3.36 - 4.12, 4.13-4.89 and > 4.90 mmol/L. Additionally, in order to improve comparisons between the methods the samples were stratified according to cut-off points recommended by the NCEP ATP III for TC levels (≤4.13, 4.14 - 5.16, 5.17 - 6.20, 6.21 - 7.24 and ≥7.25 mmol/L) and for TG levels (≤1.13, 1.14-1.69, 1.7 - 2.25, 2.26-2.82, and 2.83-4.52 mmol/L).

### Statistical analysis

Distribution of TC, TG, HDL-C, D-LDL-C, F-LDL-C, A-LDL-C and S-LDL-C was normal according to Kolmogorov-Smirnov test. Differences between values calculated with different formulas and from direct method were examined by the Student's paired t test. The Passing-Bablok linear regression was used to evaluate the degree of association between LDL-C values from different formulas and from the direct method [14]. Two-tailed P values less than 0.05 were considered statistically significant. Statistic analyses were conducted using Microsoft^® ^Office Excel 2003.

## Results

Lipoprotein concentrations and their distributions in the initial group are given in Table 1.

**Table 1:** Basic serum lipoprotein measurements, their distributions and mean percentage differences in the initial group (n = 1010)

	TC, mmol/L	TG, mmol/L	HDL-C, mmol/L	D-LDL-C, mmol/L	F-LDL-C, mmol/L	ΔF-LDL-C, %	A-LDL-C, mmol/L	ΔA-LDL-C, %
Mean	5.79	1.88	1.31	3.9	3.63*	-6.9	3.72*	-3.9
SD	1.21	0.92	0.34	1.09	1.06	8.8	1.04	14.6
1st quartile	4.93	1.14	1	3.2	2.89	-12.1	2.99	-13.8
Median	5.7	1.68	1.2	3.8	3.55	-6.5	3.66	-6.3
3rd quartile	6.5	2.41	1.5	4.5	4.27	-1.8	4.39	5

* p < 0.001

TC: total cholesterol, TG: triglycerides, HDL-C: high-density lipoprotein cholesterol, LDL-C: low-density lipoprotein cholesterol, D-LDL-C: directly measured LDL-C, F-LDL-C: LDL-C calculated by Friedewald's formula, ΔF-LDL-C: mean percentage difference for Friedewald's formula, A-LDL-C: LDL-C calculated by Anandaraja's formula, ΔA-LDL-C: mean percentage difference for Anandaraja's formula.

The Student's paired t-test showed that D-LDL-C values were significantly higher than F-LDL-C and A-LDL-C values (p < 0.001). Directly measured LDL-C concentrations exceeded F-LDL-C and A-LDL-C concentrations in 82% and 65% of samples, respectively. The mean absolute bias and the mean %ΔLDL between calculated LDL-Cs compared to the direct method were - 0.27 ± 0.31 mmol/L and -6.9 ± 8.8% for Friedewald's formula and -0.18 ± 0.51 mmol/L and -3.9 ± 14.8% for Anandaraja's formula.

Mean percentage differences between Friedewald's formula and direct LDL-C values (%ΔF-LDL-C) were negative in all quartiles, the lowest in the first, which suggested that the whole distribution was shifted to negative values (Table 1). Mean percentage differences between Anandaraja's formula and direct LDL-C values (%ΔA-LDL-C) were negative in the first quartile but positive in the third and the whole distribution was also shifted towards negative values (Table 1).

A comparison of D-LDL-C (x) versus F-LDL-C (y) and D-LDL-C (x) versus A-LDL-C(y) values resulted in the following regression equations: y = -0.17 + 0.980×, r = 0.96 and y = 0.129 + 0,971×, r = 0.89, respectively (data not shown). In the whole initial group Friedewald's formula correctly classified 65% of the subjects and Anandaraja's formula only 55%. The same percentage of subjects (31%) was underestimated by both formulas (data not shown).

### Derivation of the modified formula

The obtained unsatisfactory results led us to re-examine Friedewald's formula for LDL-C estimation. Following the procedure which led to Friedewald's formula derivation we re-calculated factor for VLDL-C concentration estimation. We used TC, TG, LDL-C and HDL-C concentration measurements in the initial group to calculate the VLDL-C/TG ratio for a Serbian population. We first subtracted the sum of HDL-C and LDL-C from TC for each person. This was estimation of VLDL-C concentration for each person. Thereafter, we divided the particular TG concentration with the corresponding calculated VLDL-C to determine the mean of the ratio. The TG/VLDL mean ratio was 3 compared with 2.2 according to Friedewald [8]. Therefore, the modified formula should be stated as follows: S-LDL-C (mmol/L) = TC - TG/3 - HDL-C. The percentage difference for our modified formula (%ΔS-LDL-C) was calculated in the same way as for Friedewald's and Anandaraja's formulas.

Our observation was validated in the population consisting of 1043 patients. Lipoprotein concentrations and their distributions for the validation group are given in Table 2.

**Table 2:** Basic serum lipoprotein measurements, their distributions and mean percentage differences in the validation group (n = 1043)

	TC, mmol/L	TG, mmol/L	HDL-C, mmol/L	D-LDL-C, mmol/L	S-LDL-C, mmol/L	ΔS-LDL-C, %	F-LDL-C, mmol/L	ΔF-LDL-C, %	A-LDL-C, mmol/L	ΔA-LDL-C, %
Mean	6.13	1.71	1.34	4.29	4.23	-0.9	4.02*	-6.1	4.09*	-3.8
SD	1.31	0.87	0.36	1.2	1.16	9.3	1.15	9.9	1.14	14.1
1st quartile	5.2	1.08	1.07	3.43	3.43	-6.3	3.22	-11.5	3.28	-12.7
Median	6.06	1.52	1.3	4.2	4.13	-2.3	3.95	-6.3	4.11	-5.3
3rd quartile	7.02	2.16	1.55	5.1	4.98	2.7	4.78	-1.3	4.82	3.9

* p < 0.001

TC: total cholesterol, TG: triglycerides, HDL-C: high-density lipoprotein cholesterol, LDL-C: low-density lipoprotein cholesterol, D-LDL-C: directly measured LDL-C, S-LDL-C: LDL-C calculated by new modified formula, ΔS-LDL-C: mean percentage difference for new modified formula, F-LDL-C: LDL-C calculated by Friedewald's formula, ΔF-LDL-C: mean percentage difference for Friedewald's formula, A-LDL-C: LDL-C calculated by Anandaraja's formula, ΔA-LDL-C: mean percentage difference for Anandaraja's formula.

A significant difference between S-LDL-C and D-LDL-C values was not found. The absolute bias between these two methods was -0.06 ± 0.37 mmol/L and the mean %ΔS-LDL-C was -0.9 ± 9.3%. A high correlation (r = 0.96) was observed between calculated and measured values.

**Figure 1:**
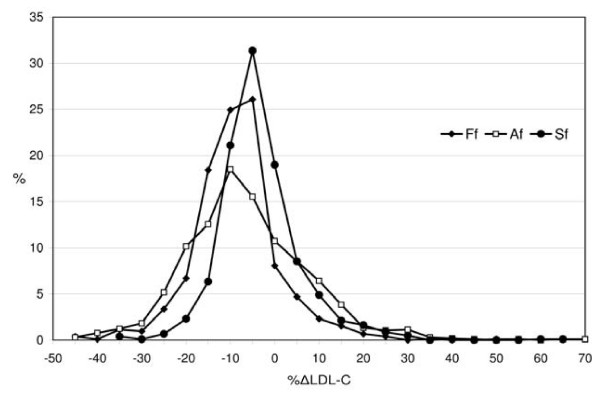
**Distribution of percentage differences for the Friedewald's, Anandaraja's and Serbian formula (Ff, Af and Sf, respectively) in the validation group**.

F- LDL-C and A-LDL-C values exhibited similar characteristics in both study groups and were compared with S-LDL-C values by calculating the percentage difference (%ΔLDL). A comparison between the values estimated by the three formulas is shown in Figure 1. The whole distribution of %SΔLDL-C values was almost symmetrical around the zero point indicating a similar number of negative and positive biases, all of which were less than those obtained by Friedewald's and Anandaraja's formulas.

Subgroups defined by cut-off values (ranges) for TC, TG and D-LDL-C values provided by NCEP ATPIII guidelines were analysed separately (Table 3). F-LDL-C was significantly lower (p < 0.001) compared to D-LDL-C in all TC, TG and D-LDL-C ranges. A-LDL-C showed no significant difference compared to D-LDL-C except for when D-LDL-C levels were less than 3.35 mmol/L. The optimal and closest results to D-LDL-C values were obtained by our modified formula (S-LDL-C) (Table 3). No differences between values were found at both the lowest and the highest TC levels (at TG concentrations 2.26-2.82 mmol/L and at LDL-C levels <4.12 mmol/L). Linear regression analyses demonstrated a high correlation of estimated LDL-Cs with the measured D-LDL-C (r > 0.91) in all TC, TG and D-LDL-C ranges.

The ability of the formulas to correctly classify subjects at the clinical decision cut-off points in specific subgroups is shown in Table 3. The percentages of samples correctly classified in risk categories for all formulas decreased with increasing TC, TG and LDL-C concentrations except at the highest concentrations of TC and LDL-C.

**Table 3:** Summary of the means ± SDs and percentages of correctly classified subjects in risk categories with regard to the TC, TG and D-LDL-C concentrations given by NCEP ATP III in the validation group

						% of subjects properly classified by the following formulas		
						
TC, mmol/L	n	D-LDL-C, mmol/L	F-LDL-C, mmol/L	A-LDL-C, mmol/L	S-LDL-C, mmol/L	Friedewald	Anandaraja	Modified
≤4.13	55	2.34 ± 0.44	2.13 ± 0.45**	2.12 ± 0.45**	2.28 ± 0.39	82%	84%	86%
4.14-5.16	192	3.1 ± 0.46	2.86 ± 0.43**	2.89 ± 0.41**	3.04 ± 0.39*	59%	52%	75%
5.17-6.20	310	3.88 ± 0.48	3.60 ± 0.41**	3.73 ± 0.43**	3.80 ± 0.38**	54%	42%	64%
6.21-7.24	287	4.78 ± 0.59	4.46 ± 0.43**	4.54 ± 0.42**	4.69 ± 0.39**	52%	47%	63%
≥7.25	199	5.92 ± 0.85	5.68 ± 0.77**	5.71 ± 0.70**	5.92 ± 0.77	92%	87%	94%
TG, mmol/L								
≤1.13	280	3.83 ± 0.99	3.76 ± 0.97**	4.05 ± 1.04**	3.86 ± 0.98*	74%	59%	80%
1.14-1.68	324	4.32 ± 1.14	4.08 ± 1.08**	4.16 ± 1.08**	4.24 ± 1.08**	63%	59%	70%
1.69-2.25	217	4.58 ± 1.22	4.21 ± 1.21**	4.16 ± 1.21**	4.45 ± 1.20**	59%	54%	73%
2.26-2.82	97	4.64 ± 1.32	4.24 ± 1.41**	4.15 ± 1.35**	4.54 ± 1.41	55%	49%	65%
2.83-4.52	125	4.52 ± 1.32	3.98 ± 1.30**	3.85 ± 1.24**	4.4 ± 1.29*	49%	52%	67%
LDL-C, mmol/L								
≤2.59	64	2.21 ± 0.30	2.07 ± 0.44**	2.23 ± 0.61	2.27 ± 0.39	91%	77%	81%
2.6-3.35	181	3.03 ± 0.21	2.90 ± 0.37**	3.00 ± 0.50	3.07 ± 0.35	61%	55%	71%
3.36-4.12	262	3.77 ± 0.22	3.60 ± 0.45**	3.71 ± 0.57*	3.79 ± 0.44	61%	45%	69%
4.13-4.89	213	4.50 ± 0.23	4.15 ± 0.42**	4.25 ± 0.56**	4.37 ± 0.39**	52%	49%	63%
≥4.9	323	5.67 ± 0.74	5.29 ± 0.81**	5.28 ± 0.81**	5.23 ± 0.81**	67%	66%	80%

* p < 0.05, ** p < 0.001

LDL-C: low-density lipoprotein cholesterol, D-LDL-C: directly measured LDL-C, F-LDL-C: LDL-C calculated by Friedewald's formula, A-LDL-C: LDL-C calculated by Anandaraja's formula, S-LDL-C: LDL-C calculated by the new modified formula.

**Figure 2:**
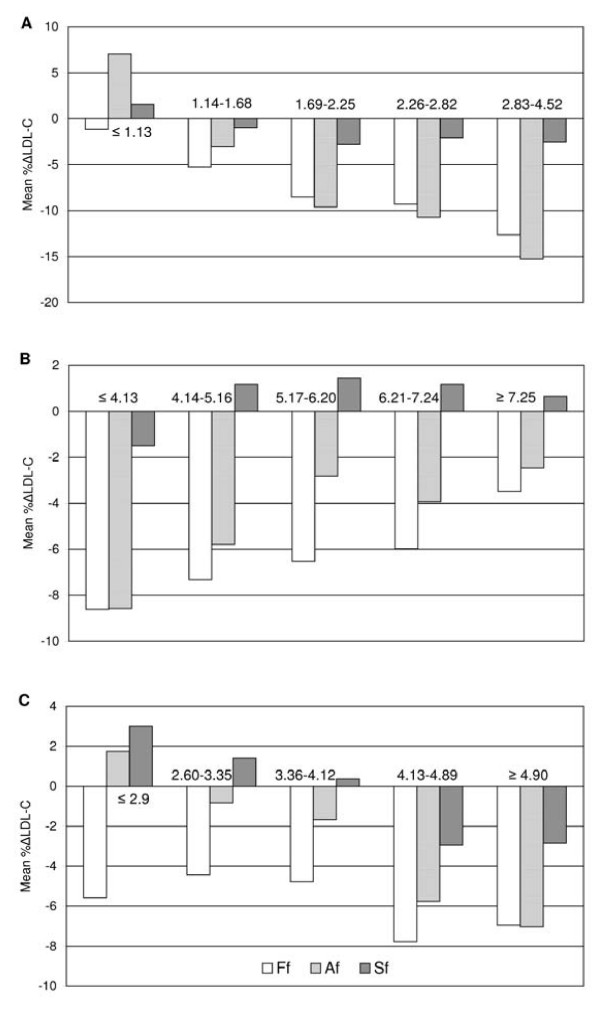
**Mean percentage differences for Friedewald's (Ff), Anandaraja's (Af) and our modified (Sf) formula according to TC (A), TG (B) and LDL-C (C) cut-off values proposed by NCEP ATP III in the validation group**.

Mean %ΔLDL-C values steadily increased with increasing TG concentrations but decreased with increasing TC and D-LDL-C concentrations. Mean %ΔF-LDL-C values were negative in all ranges of TC, TG and D-LDL-C. Mean %ΔA-LDL-C values were negative in all ranges of TC but less than the same values obtained by Friedewald's formula. When TG concentrations were less than 1.14 mmol/L and D-LDL-C concentrations were less than 2.59 mmol/L mean %ΔA-LDL-C values were positive (Figure 2).

Figure 2 shows that mean %ΔS-LDL-C values were not different than ± 3% in whole TC, TG and D-LDL-C ranges and they were much lower than mean %ΔLDL-C values obtained from the other two formulas. This suggests a better agreement with D-LDL-C than that obtained from the two other formulas.

When TG concentrations were ≤1.13 mmol/L and between 2.83-4.52 mmol/L our modified formula led to the highest percentages of individuals having LDL-C values falling within ± 10%ΔLDL-C (88% and 60%, respectively) compared to Friedewald's (87% and 37%, respectively) and Anandaraja's (60% and 32%, respectively) (data not shown). In the whole validation group %ΔLDL-C differed by more than ± 10% in 20%, 36% and 55% of the subjects when our modified, Friedewald's and Anandaraja's formulas were employed, respectively.

## Discussion

Strategies for treatment of lipid abnormalities are primarily based on LDL-C concentration. Therefore, LDL-C must be accurately determined to establish a personal CHD risk profile in order to initiate dietary adjustments, drug therapy and to monitor their effects [4].

In the past few decades attempts have been made to derive more accurate formulas for LDL-C calculation than the widely used Friedewald's formula [15-20]. Although the newer formulas offered few advantages over the Friedewald's, they have performed only marginally better, possibly due to diversity in terms of study populations and/or pathologies [21-23]. Some of them included apolipoprotein concentrations, apoA-I and/or apoB [18-20]. Anandaraja and colleagues [12] described a new formula for LDL-C calculation in an Indian population of 1000 patients by applying multiple linear regression analysis and validated its accuracy in 1008 patients. In their study the mean LDL-C concentrations measured by a precipitation method and by their formula were 3.04 ± 1.04 mmol/L and 2.96 ± 0.96 mmol/L, respectively. The mean absolute difference between both methods was 0.1 ± 0.24 mmol/L and good correlation was found (r = 0.97). In addition, they confirmed a reduction in the false overestimation of LDL-C compared with Friedewald's formula. Anandaraja and colleagues called for the reliability of their formula to be tested in other populations.

On the other hand, Friedewald's formula has been shown to be relatively reliable and recommended by the NCEP as a routine method [5] for estimation of LDL-C despite it having several well-established constraints. It cannot be applied to samples containing TG levels > 4.52 mmol/L (400 mg/dL), to non-fasting samples and to samples of patients with dysbetalipoproteinemia (Fredrickson Type III) [8,13]. Some authors have demonstrated that the formula should not be used in certain groups of patients with diabetes, liver and renal dysfunction even with TG levels < 4.52 mmol/L [17,22,23]. The formula relies on the accuracy of the TC, TG and HDL-C assays and also on an additional mathematical term that is used to estimate the VLDL-C concentration. It assumes a fixed relationship between TC, TG, and HDL-C in fasting serum providing the TG/cholesterol ratio in the VLDL is constant and the assumption that TG is only present as VLDL. As well, the homogenous Roche method we have used has some limitations [6] although it has been reviewed by Nauck et al [13] to be precise and acceptably accurate. It gives an improvement in the measurement of LDL-C in samples with high TG and may assist better in classification of patients at risk categories for cardiovascular diseases than Friedewald's equation.

Anandaraja's team did not propose any limitations to their formula. Comparing the mean value of the direct LDL-C obtained in the first 1000 patients and that in the validation group of 1008 patients it seemed they did not exclude samples with high TG levels [12]. In a study of over 10000 Brazilian patients Gasko and colleagues [24] supported Anandaraja's formula. The mean LDL-C level measured by a direct method and that estimated by the new formula were similar to the Indian population (2.99 ± 0.57 mmol/L and 2.97 ± 0.59 mmol/L, respectively). The correlation coefficient between both methods was r = 0.97. Anandaraja's formula was also checked in 230 Greek patients (118 had metabolic syndrome and 112 were healthy) by Gazi and Elisaf [25]. Friedewald's and Anandaraja's formulas gave similar results in the examined Greek population. The latter was approved for use in their laboratories.

In our study we investigated if Anandaraja's formula could be applied in the Serbian population by comparing the value obtained with that of the homogenous direct method for LDL-C determination. This is the first study of its kind where the reliability and accuracy of Friedewald's formula were tested in the Serbian population. In our initial group LDL-C values from the direct measurement and from Anandaraja's formula were both higher than the values in Indian, Brazilian and Greek populations by almost 1 mmol/L [12,24,25]. The A-LDL-C concentration was significantly lower than the D-LDL-C concentration (Table 1). The correlation coefficient between methods was good (r = 0.89) but lower than previously published (r = 0.97) [12,24].

To the best of our knowledge only Paz and colleagues [26] have performed a detailed systematic analysis of the reliability of Anandaraja's formula. They tested the new formula in schizophrenic patients treated with antipsychotic drugs. Their results demonstrated that LDL-C_Anandaraja _concentrations were underestimated or overestimated compared to LDL-C_Electrophoresis _and depended on the HDL-C concentrations. They found a higher correlation and a lower estimation error between LDL-C_Electrophoresis _and LDL-C_Friedewald _than LDL-C_Electrophoresis _and LDL-C_Anandaraja_. For that reason improved accuracy of Anandaraja's formula over Friedewald's formula was not claimed. Data from our study are in agreement with Paz and colleagues. We employed two apparently healthy populations from two different Serbian cities in which all analyses were completed with the same type of reagents on the same class of autoanalyser. In both populations the results were very similar and did not support Anandaraja's formula (Table 1 and 2). The percentages of patients properly classified in NCEP's risk categories and the percentage of patients that fell in the ± 10%ΔLDL-C group were smaller when compared to Friedewald's formula in all ranges of TC, TG and LDL-C (Table 3).

Figure 1 shows that %ΔLDL-C distribution for Anandaraja's formula was overrached and dismounted compared to Friedewald's. Our findings concerning Friedewald's formula are consistent with other published studies. As reported earlier [10,27,28], we found that calculated LDL-C values derived from Friedewald's formula often underestimate directly measured LDL-C concentrations. Tighe and colleagues [10] found good correlation between LDL-C calculated by Friedewald's formula and directly measured LDL-C (r = 0.90). Only 48.1% of samples in Tighe's study showed similar results compared to 63% in our validation group.

Jun and co-workers [27] revealed that F-LDL-C differed significantly from D-LDL-C over the concentration ranges of both TC and TG. They found that the mean %ΔLDL was -9.1% and assumed that this difference was critical for the evaluation of patients with hyperlipidemia. Their study demonstrated that higher TG resulted in a greater %ΔF-LDL-C and increased TC was associated with decreased %ΔF-LDL-C, which was also confirmed in our current study.

It would appear that calculated LDL-Cs by both Friedewald's and Anandaraja's formulas give unsatisfactory results compared to the direct homogenous method. It seems that the only advantage of Anandaraja's formula is the requirement of the concentration of only 2 variables (TC and TG) that reduces the analytical error. Our study in a Serbian population revealed that the HDL-C concentration should not be omitted from the formula, in agreement with Paz and colleagues [26].

In the course of the present study to investigate the reliability of Friedewald's and Anandaraja's formulas we derived a new modified formula. It resembles Friedewald's and it is based on the original study [8]. Simple division of plasma TG by 2.2 for mmol/L or 5 for mg/dL does not give a very accurate estimation of VLDL-C even in a healthy population. Some authors have proposed alternative calculations including TG/4, TG/4.5, TG/5, TG/5.5, TG/6, TG/7 and TG/8 (mg/dL) [15,16]. Nakanishi and colleagues [16] demonstrated that the TG/5 formula correlated well with measured LDL-C and had the smallest mean difference between estimated and measured LDL-C in middle-aged Japanese men. Gonzales-Estrada [29] concluded that the DeLong (TG/2.7 mmol/L or TG/6.17 mg/dL) calculation [15] was more convenient than the original Friedewald's formula for most cases, despite a high error. On the contrary, according to some authors DeLong's formula did not improve LDL-C determination [21,23].

In our initial population we calculated that to determine cholesterol in VLDL, TG should be divided by 3 (mmol/L) or 6.85 (mg/dL). Our modified formula was tested and its accuracy was validated in our second population. S-LDL-C showed no statistically significant difference compared to D-LDL-C in the validation group, despite the fact that there was still an underestimation. No significant difference between these two mean values was found in situations of TC < 4.13 mmol/L and TC > 7.24 mmol/L, TG 2.26 - 2.82 mmol/L and LDL-C < 4.12 mmol/L. The mean %ΔS-LDL-C was -0.9%, the smallest among these three calculations (Table 2). Our modified formula exhibited the lowest mean %ΔLDL-C in all ranges of TG, TC and D-LDL-C compared to Friedewald's and Anandaraja's formulas (Figure 2). As for the accuracy of the calculation method, the proportion of samples falling within a fairly broad ± 10%ΔLDL-C range was the highest. Our results indicate that LDL-C concentrations derived from the modified formula provide more accurate results compared to those derived from Friedewald's and Anandaraja's formulas for Serbian population. 

According NCEP-ATP III guidelines, LDL-C concentrations < 3.36 mmol/L are considered desirable while those > 4.14 mmol/L are considered high. Medication should be administered to subjects falling into the latter group [4]. Our study demonstrated that 45%, 45% and 26% of samples from Friedewald's, Anandaraja's and our modified formula, respectively underestimated the diagnostic LDL-C level of 4.14 mmol/L and were classified one cut-off point below that indicated for therapy (data not shown).

In conclusion, regarding patients' convenience, financial reasons and precision and accuracy we propose that our new modified formula should be used instead of Friedewald's formula for the estimation of LDL-C concentration in the Serbian population. We appeal to all laboratories principally in Serbia and in neighbouring countries with similar living conditions and habits to test our modified formula before its eventual implementation.

## List of abbreviations

CHD: coronary heart disease; NCEP: National Cholesterol Education Program; ATP III: Adult Treatment Panel III; F-LDL-C: LDL-C value calculated by Friedewald's formula; A-LDL-C: LDL-C value calculated by Anandaraja's formula; S-LDL-C: LDL-C value calculated by our new modified formula; %ΔLDL: percentage difference.

## Competing interests

The authors declare that they have no competing interests.

## Authors' contributions

AV wrote the manuscript and statistically analyzed data. JKS participated in the study design, statistically analysed data and critically revised the manuscript, SS carried out all aspects of the study design, statistically analysed data and critically revised the manuscript, NB performed experimental work and collected samples, MV performed experimental work and collected samples, JM performed experimental work and collected samples, VSK critically revised the manuscript, AZ critically revised the manuscript, DP collected samples. All authors read and approved the final manuscript.
